# Enhancing equity and diversity in palliative care clinical practice, research and education

**DOI:** 10.1186/s12904-023-01185-6

**Published:** 2023-06-05

**Authors:** Jonathan Koffman, Gilla K. Shapiro, Christian Schulz-Quach

**Affiliations:** 1 Hull York Medical School , Wolfson Palliative Care Research Centre, University of Hull, Hull, HU6 7RX UK; 2 Division of Psychosocial Oncology, Department of Supportive Care, Princess Margaret Cancer Centre, University Health Network (UHN), Toronto, Canada; 3 Department of Psychiatry, University of Toronto, Toronto, Canada; 4 Social and Behavioural Health Sciences Division, Dalla Lana School of Public Health, University of Toronto, Toronto, Canada

## Abstract

Health disparities in palliative care are preventable consequences of structural discrimination and marginalization. The first step in addressing a problem is recognizing there is one and devotion to fully understanding its multifaceted nature. Palliative care clinicians, educators and researchers must prioritize investigating and mitigating the effects of racial, social, and intersectional injustice.

## Main text

Health disparities are the preventable consequences of structural discrimination and marginalization that, if left unaddressed, lead to and perpetuate substantial preventable morbidity and mortality [1]. When it comes to the delivery of high-quality palliative care, there is now increasing evidence of systematic and structural health disparities that stymie the possibility of achieving what people refer to as a "good death" [2–4] and contribute to prolonged or complicated grief among caregivers [5]. There are notable disparities in accessing palliative care among minority groups as well as across global settings [6].

Health equity is recognized by the World Health Organisation as a vital component of social justice and is described as the absence of unfair or avoidable differences [6]. The Worldwide Hospice Palliative Care Alliance has recently called out palliative care as "one of the most inequitable areas of healthcare" and emphasized the need to improve equitable access to palliative care [7]. Equity is recognized to be a key component of palliative care and is achieved when all can reach their fullest health potential through timely, appropriate, and high-quality care [8]. Diversity considers what makes each person unique in their illness narrative in terms of heritage, sociodemographic characteristics, and identity expression including a thorough reflection of intersectional identities [9]. Palliative care services that relieve suffering and improve quality of life must be provided to *all* people irrespective of who they are, where they live, or their life experiences.

While this is not a new phenomenon, recent global movements have emphasized societal fissures that demand attention [10]. Among many other sociopolitical and environmental issues, international movements like Black Lives Matter [11], national scandals like the residential schools scandal in Canada and the “Windrush” scandal in the UK [12], the disproportionate impact of the COVID-19 pandemic on those living on the margins of society, who already experienced multiple disadvantages [13], global discrimination of those who are 2SLGBTQIA + [14, 15], have underscored societal fissures and inequities that demand attention. It is also important to recognize that future environmental and political events, such as climate change, war and disasters, may compound inequalities in accessing palliative care. All have particular emphasis on end-of-life care; inequities in life are mirrored in the experience of dying.

**Fig. 1:**
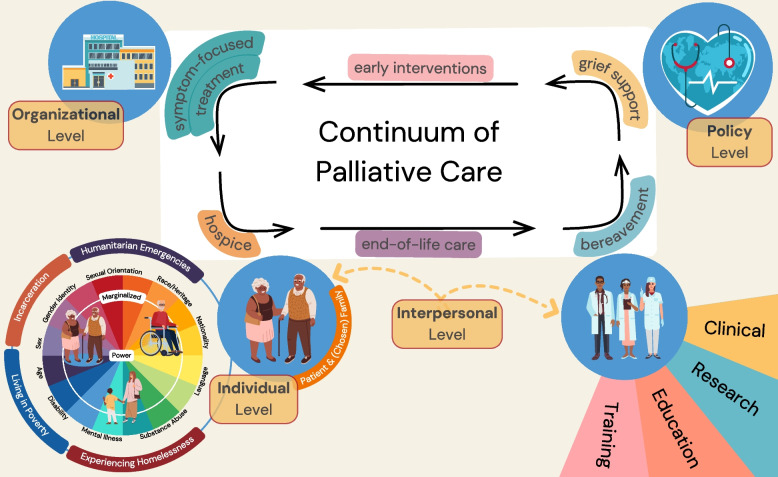
Levels of health equity impact in palliative care (i.e., individual, interpersonal, organizational, policy-related). Structural discrimination and marginalization in the interplay of those four levels affect the quality of palliative care services along its continuum

Enhancing equity and diversity in palliative care practice will require a person-centred approach with community support that advocates for change on multiple levels: individual (patient and caregiver), interpersonal (health care team), organizational, and policy (Fig. 1). Equitable access is required across the entire continuum of palliative care services that begins with early interventions, continuing with symptom-focused treatment, hospice, and end-of-life care, as well as bereavement and grief support. Reducing disparities in palliative care must be concerned with the clinical practice of palliative care, as well as diversity, inclusion and access in education, training, and research. In education and training, the discipline of palliative care must take an active role in recruiting and teaching diverse clinicians, nationally and internationally. In terms of equity in palliative care research, there is a need to develop diverse research teams, partner with community groups and patients with lived experience, and enable the democratic expression of ideas in research teams who are involved in the production and review of new knowledge.

The first step in addressing a problem is recognizing there is one and devotion to fully understanding its multifaceted nature. Palliative care clinicians, educators and researchers must prioritize investigating and mitigating the effects of racial, social, and intersectional injustice. This collection on 'Equity and diversity in palliative care" (https://www.biomedcentral.com/collections/EDPC) therefore welcomes contributions from clinical research as well as original theoretical reflections relating to philosophical, ethical, and policy issues specific to this theme to mitigate the effects of racial, social and intersectional injustice. We actively encourage critical self-reflection and acknowledgement of conscious and implicit bias that has shaped the research questions we ask, how we address them, the way we report study findings, and the composition of research teams.

## Data Availability

Not applicable.

## Abbreviation

2SLGBTQIA +: Two-Spirit, Lesbian, Gay, Bisexual, Transgender, Queer, Intersex, Asexual, and ( +) any gender identities or sexual orientations not included in this acronym
